# Wheat fiber-induced peripheral regulatory T-cells suppress development of colitis

**DOI:** 10.1016/j.mucimm.2025.12.003

**Published:** 2025-12-22

**Authors:** Seong-eun G. Kim, Hirohito Abo, Yanling Wang, Shawn Winer, Daniel A. Winer, Michael Pellizzon, Vu L. Ngo, Andrew T. Gewirtz

**Affiliations:** a Center for Inflammation, Immunity and Infection, Institute for Biomedical Sciences, Georgia State University, Atlanta, GA 30303, United States; b Department of Laboratory Medicine and Pathobiology, University of Toronto, Toronto, ON, Canada; c Buck Institute for Research on Aging, Novato, CA, United States; d Division of Cellular & Molecular Biology, Diabetes Research Group, Toronto General Hospital Research Institute (TGHRI), University Health Network, Toronto, ON M5G 1L7, Canada; e Department of Immunology, University of Toronto, Toronto, ON M5S 1A8, Canada; f Research Diets, Inc., New Brunswick, NJ, United States

**Keywords:** Inflammatory bowel disease, Dietary fiber, Microbiota, Metabolites

## Abstract

Reduced dietary fiber intake is associated with, and may have contributed to, the post-mid-20th century increase in immune-mediated chronic inflammatory diseases, including inflammatory bowel disease (IBD). Reduced fiber intake has resulted, in part, from increased consumption of highly refined foods including those made from white flours, generation of which involves removal of much of the fiber naturally present in wheat kernels. Accordingly, we hypothesized that wheat fiber (WF) might protect against chronic inflammatory diseases. We tested this notion in a murine T-cell-transfer colitis model. *Rag1*^*−/−*^ mice were fed purified (open-source) low-fiber diets enriched, or not, with WF and then administered CD45Rb^hi^ T-cells. WF conferred robust protection in this colitis model as assessed by an array of clinical, histopathologic, morphologic, and immune-related parameters. WF’s protection against colitis associated with a microbiota-dependent increase in Foxp3^+^ T-cells (Tregs), which could be recapitulated in vitro. WF did not induce Tregs in mice lacking conserved non-coding sequence 1 knock-out (*CNS1*), which is known to drive peripheral Treg development, nor did WF protect against T-cell-transfer colitis driven by transplant of colitogenic T-cells from *CNS1*^*−/−*^ mice. Thus, enriching diet with WF has potential to promote microbiota-dependent peripheral Treg development and, consequently, protect against chronic inflammatory diseases.

## Introduction

Intertwined changes in food production methods and dietary habits have resulted in decreased consumption of fiber in a manner that roughly parallels the increasing prevalence of immune-mediated chronic inflammatory diseases, including inflammatory bowel disease (IBD).^1–3^ This correlation, combined with a large body of epidemiological evidence that fiber intake is associated with good health, and the fact that dietary fiber directly interacts with, and is metabolized by, gut microbiota, which is increasingly recognized to be a key chronic inflammatory disease determinant, has led to the hypothesis that fiber consumption protects against an array of diseases including IBD. In accord with this notion, maintaining mice on low-fiber diets, rather than the standard fiber-rich grain-based chow diets that lab mice are typically fed, increases severity of colitis in both acute (DSS) and T-cell transfer colitis models.^4^ Subsequent attempts to ameliorate colitis severity by enrichment of low-fiber diets with specific semi-purified fibers found fiber- and model-specific impacts. For example, inulin, which is readily fermentable to butyrate, which is known to induce regulatory T-cells (Treg), provides modest protection in a T-cell mediated colitis model^5^ but markedly exacerbates DSS-induced colitis.^6^ In contrast, psyllium, which is only minimally fermentable, confers strong protection in both the DSS and T-cell transfer model.^4^ Psyllium’s protection is mediated activation of the FXR bile acid receptor, while inulin’s exacerbation of DSS colitis occurred despite it increasing bile acid levels and activating this receptor^7^.

The rationale for our initial focus on these particular fibers is that many humans seek to improve their health by consuming them in supplements. However, their dramatically differing impacts on microbiota and colitis severity led us to appreciate the importance of also examining impacts of fibers naturally present in foods, especially those that became less frequently consumed as the prevalence of IBD, and other chronic inflammatory disease, increased. Hence, this study and a sister study performed in parallel,^8^ examined impacts of wheat fiber (WF) on murine colitis. Wheat kernels, particularly the bran portion, are rich in fiber such that “whole wheat” or “whole-grain” breads can approach 15% fiber by weight. The majority of this fiber is absent in “white” breads, which are made from the endosperm portion of the wheat kernel. Thus, we compared the consequence, of maintaining mice on low-fiber diets enriched, or not, with WF. Our parallel study found that WF protected against DSS colitis via an FXR-independent microbiota-dependent macrophage-mediated mechanism^8^. Here, we report that WF protected against T-cell transfer colitis via microbiota-mediated fermentation-independent promotion of peripheral Treg development.

## Results

### Wheat fiber protects against T cell-mediated colitis

**Fig. 1.**
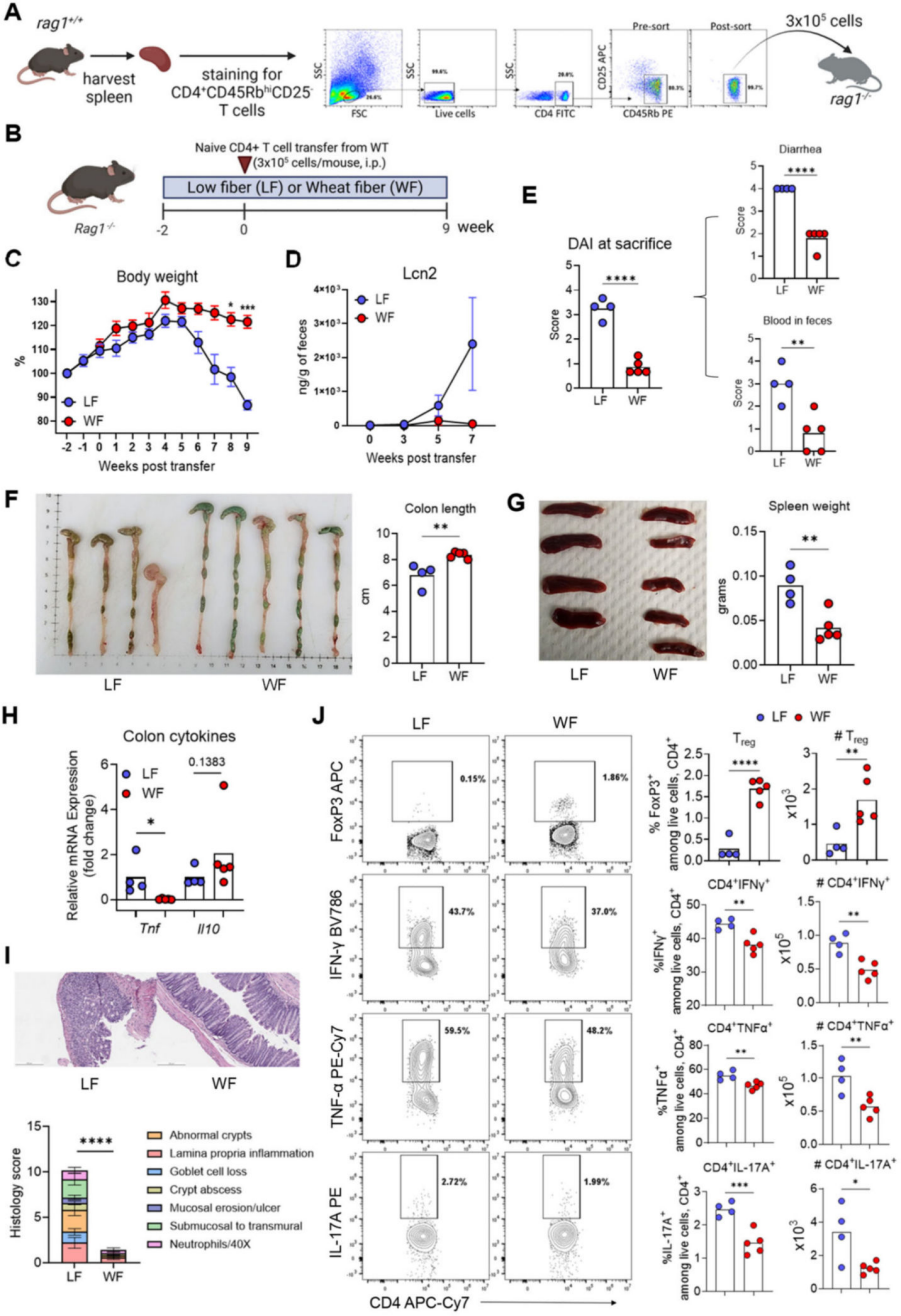
Wheat fiber protected mice against T-cell transfer colitis. *Rag1*^*−/−*^ mice (n = 5 per group) were fed either a low-fiber (LF) or wheat fiber-enriched (WF) diet for 2 weeks and received 3×10^5^ naïve CD4^+^ T cells from WT mice. (A) Gating strategy and purity of the transferred cells. (B) Schematic. (C) Body weight. (D) Fecal Lcn2 levels measured by ELISA. (E) Disease activity index. (F-G) Colon length and spleen weight at sacrifice. (H) Colon pro-inflammatory cytokines assessed by RT-qPCR. (I) Representative colon histology and scoring. (J) Colon lamina propria CD4^+^ T cells analyzed by flow cytometry. Results are representative of three independent experiments using both male and female mice. Statistical significance was assessed using two-way ANOVA followed by Sidak’s multiple comparisons (B) or unpaired two-tailed *t* test (C-J). *P < 0.05, **P < 0.01, ***P < 0.001, ****P < 0.0001.

To probe if wheat fiber (WF) impacted the severity of T-cell-mediated colitis, *Rag1*^*−/−*^ mice, raised on grain-based chow (GBC; LabDiet Cat# 5001), were fed a low-fiber (LF) compositionally defined or a WF-enriched version of this diet (Table 1). Two weeks later, such mice were administered CD4^+^CD25^-^CD45Rb^hi^ T (i.e. naïve) cells, which had been freshly isolated from GBC-fed WT mice (Figs. 1A and B). Body weight and fecal lipocalin-2 (Lcn2) levels, which reflect intestinal inflammation,^9^ were monitored. LF-fed mice exhibited indices of colitis, including weight loss and elevated fecal Lcn2 expression by 5 weeks post-T cell transfer (Fig. 1C and D). A few weeks later, one LF-fed mouse reached endpoints (euthanized at 7 weeks post transfer).WF-fed mice exhibited only modest changes in these parameters, suggesting WF protected against colitis in this model. Post-euthanasia analysis confirmed this notion. Specifically, compared to LF-fed mice, WF-fed mice had significantly lower DAI scores (Fig. 1D), exhibited longer colons (Fig. 1F), smaller spleens (Fig. 1G), reduced colon *Tnfα* expression (Fig. 1H), and starkly lower histopathologic disease scores (Fig. 1I). Enrichment of diet with WF also impacted the developmental fate of the transferred CD4 T cells. Specifically, WF-fed mice displayed lower levels of colonic lamina propria-derived IFNɣ^+^, Il-17A^+^, and TNFα^+^ CD4 T cells and increased levels of FoxP3^+^ regulatory T cells (Tregs) (Fig. 1J), all of which might have contributed to the reduced disease exhibited by WF-fed mice.

**Fig. 2.**
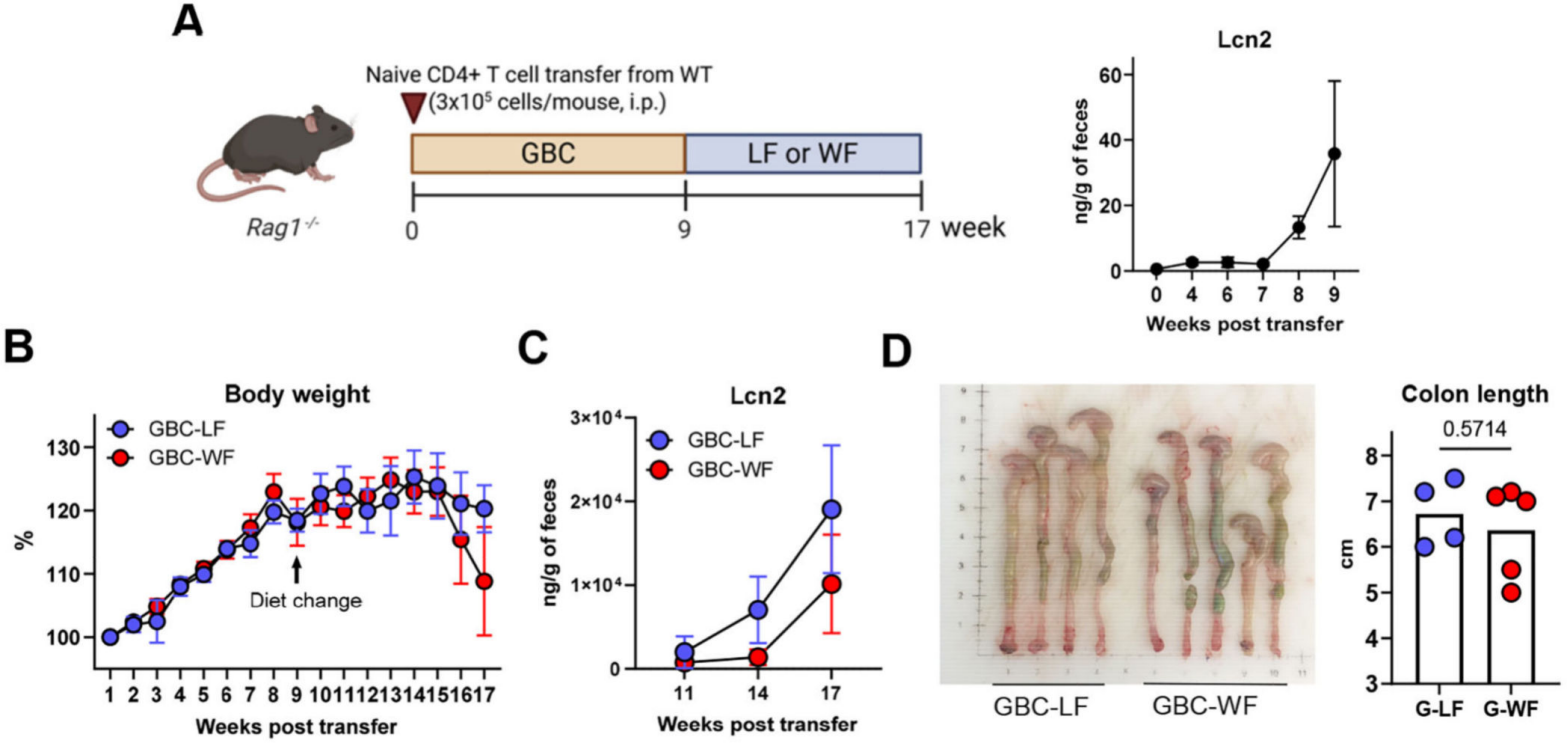
Wheat fiber did not ameliorate T-cell colitis following disease onset. *Rag1*^*−/−*^ mice (n = 5 per group, a mouse from LF group euthanized at week 11 post transfer) were injected with naïve CD4^+^ T cells while on GBC diet, then switched to either a LF or WF diet. (A) Experimental design schematic. (B) Body weight and fecal Lcn2 levels post-transfer before diet change. (C) Fecal Lcn2 levels after diet change (D) Colon length at sacrifice. Results are representative of two independent experiments. Statistical significance was assessed using unpaired two-tailed *t* test (D).

We also examined the extent to which WF might impact colitis in mice with established, but early-stage, T-cell-mediated colitis. GBC-fed *Rag1*^*−/−*^ mice were administered CD4^+^CD25^-^CD45Rb^hi^ T cells. Such mice were then monitored by measuring body weight and fecal Lcn2 levels. Modest but significant increases in fecal Lcn2 and decreases in body weight were observed by 9-weeks post T-cell transplant suggesting onset of colitis. At this time, mice were switched from GBC to the LF or WF diet (Fig. 2A). In accord with our previous study,^4^ body weights appeared to stabilize in both groups, potentially reflecting that the dietary change resulted in a rapid change in microbiota-derived T-cell antigens. In any case, body weights of both groups resumed decreasing a few weeks later at which point WF-fed mice developed colitis at least as severe as LF-fed mice as evidenced by all parameters assayed (Fig. 2B–D). Collectively, these results accord with the possibility that WF consumption protects against development of T-cell-mediated colitis but may not be able to mitigate it once it is established.

### Wheat fiber increases regulatory T cells in a microbiota-dependent manner

**Fig. 3.**
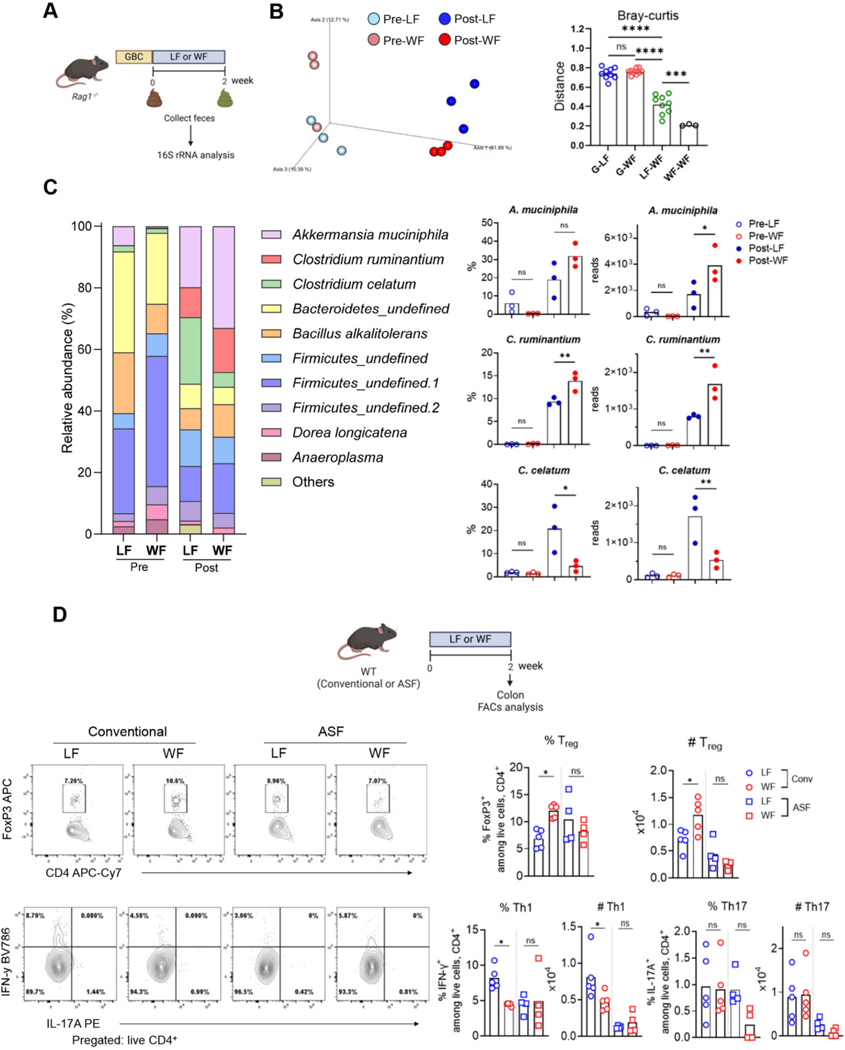
Wheat fiber increased regulatory T cells in a microbiota-dependent manner. 16S rRNA sequencing was performed on feces of *Rag1*^*−/−*^ mice (n = 3/group) fed either LF or WF for 2 weeks. (A) Schematic (B) Beta diversity (Jaccard) analysis (C) Taxonomic composition at the species level and three species altered by wheat fiber. Colonic lamina propria CD4 + T cells from different mice on the indicated diets were measured by flow cytometry (D). Regulatory T cells, Th1, and Th17 cells in conventional (n = 5/group) or ASF mice (n = 4/group) fed either LF or WF for 2 weeks. Results are representative of two independent experiments using both male and female mice. Statistical significance was assessed using one-way ANOVA followed by Tukey’s multiple comparisons test (B) or Sidak’s multiple comparisons test (C and D). ns P > 0.05, *P < 0.05, **P < 0.01, ***P < 0.001, ****P < 0.0001.

Many impacts of dietary fiber, including WF’s protection against DSS colitis, are associated with, and require, alterations in gut microbiota,^8^ prompting us to examine how dietary changes impacted the microbiotas of *Rag1*^*−/−*^ mice prior to induction of T-cell-mediated colitis (Fig. 3A). Analogous to studies in WT mice, switching the diet of *Rag1*^*−/−*^ mice from GBC to either the LF or WF resulted in a stark change in microbiota composition (Fig. 3B). Differences in microbiota composition between LF and WF fed mice were more moderate but nonetheless PCoA analysis indicated a significant difference in overall composition and some specific taxa, including WF enrichment of *Clostridium ruminantium* and depletion of *Clostridium celatum*, as well as a trend toward increased *Akkermansia muciniphilia* (Fig. 3C) without impacting overall α-diversity (Supplementary figure 1). Such taxonomic differences between LF- and WF-fed *Rag1*^*−/−*^ mice differed somewhat from what we recently observed in WT mice^8^ potentially reflecting a role for adaptive immunity in influencing microbiota composition and/or that the *Rag1*^*−/−*^ mice were maintained in a distinct vivarium due to their immune-compromised state.

The absolute dependence of the CD45Rb^hi^ T-cell-transfer colitis model on gut microbiota^10^ precludes the use of gnotobiotic mice to examine if WF’s protection in this model requires microbiota. Thus, to gain insight into this question, we examined if WF might impact T-cell differentiation, which is known to be a major determinant of disease severity in T-cell-transfer colitis models. Specifically, we analyzed colonic CD4^+^ T-cells in mice following 2 weeks feeding with LF or WF diet. WF resulted in a significant increase in the percentage and absolute number of Tregs and a reduction in Th1 cells, while Th17 cells remained unchanged (Fig. 3D). The level of Tregs in WF-fed mice was similar to that of inulin-fed mice and exceeded those of GBC-fed mice (Supplementary Fig. 2). In contrast to inulin, which increases Tregs via SCFA generation,^11^ the WF-induced increase in Treg was not associated with increased butyrate production,^8^ arguing against a role for short-chain fatty acids. Chemical analysis of WF indicated it was 97% insoluble fiber, primarily consisting of cellulose/hemicellulose.^8^ Hence, we investigated if WF inducing Tregs and suppressing T cell-mediated colitis was shared by another insoluble fiber, namely cellulose. In contrast to WF, enriching LF cellulose did not increase colon Treg levels nor protect mice against T cell-mediated colitis (Supplementary Fig. 3).

WF also induced RORγt^+^ Tregs (Supplementary Fig. 3), which are a known microbiota-dependent T cell subset.^12–14^ Considering this observation, together with the finding that WF-induced microbial changes, prompted us to assess whether WF-induced changes in T-cell required microbiota. We utilized Altered Schaedler Flora (ASF) mice, which harbor a minimal eight-species microbiota^15^ that, unlike germ-free conditions, confer relatively normal mucosal immune function. WF feeding did not impact Tregs or Th1 cells in ASF mice (Fig. 3D), arguing that despite not being fermented, WF drove Treg development via a microbiota-mediated mechanism.

### WF-induced Tregs mediate its protection against T-cell mediated colitis

**Fig. 4.**
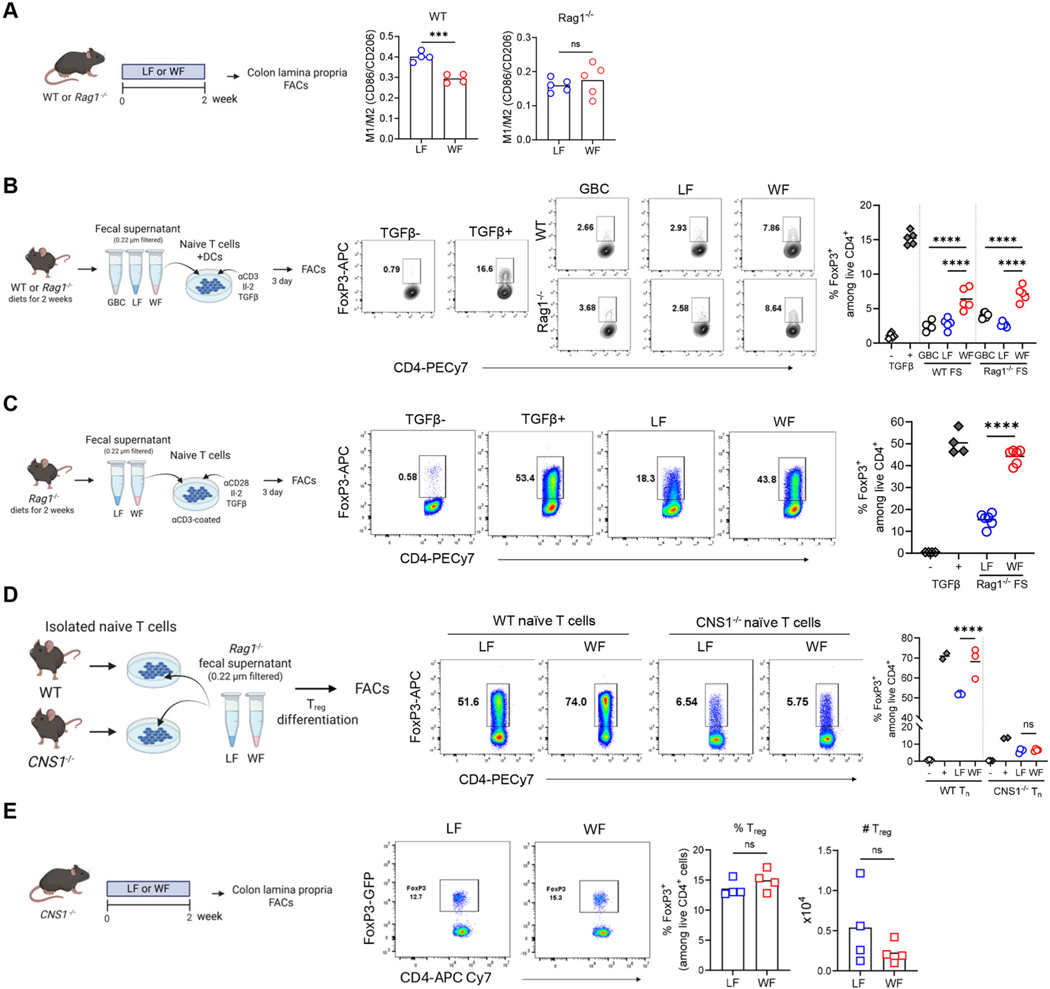
Wheat fiber-derived microbial metabolites induced peripheral regulatory T cells. (A) WT (n = 4/group) or *Rag1*^*−/−*^ mice (n = 5/group) were fed with GBC or LF or WF for 2 weeks and their colonic lamina propria macrophages were analyzed by flow cytometry. (B-D). In vitro-induced FoxP3^+^ Tregs were analyzed by flow cytometry after 3 days of culture. Splenic naïve CD4^+^ T cells (5×10^4^ cells/well) were isolated from indicated sources and stimulated under the following conditions: (B) Naïve CD4^+^ T cells and dendritic cells (10^4^ cells/well) from WT mice were cultured with soluble αCD3ε antibodies (2 μg/ml), IL-2 (15 U/ml), and TGF-β (1 ng/ml). Fecal supernatants from LF or WF-fed WT or *Rag1*^*−/−*^ mice were added. (C) Naïve CD4^+^ T cells from WT mice were cultured on αCD3ε-coated (2 μg/ml) plates with αCD28 (2.5 μg/ml), IL-2 (15 U/ml), and TGF-β (1 ng/ml). Fecal supernatants from LF or WF-fed *Rag1*^*−/−*^ mice were added. (D) Naïve CD4^+^ T cells from WT or *CNS1*^*−/−*^ mice were cultured on aCD3ε-coated (2 μg/ml) plates with αCD28 (2.5 μg/ml), IL-2 (15 U/ml), and TGF-β (1 ng/ml). Fecal supernatants from LF or WF-fed WT or *Rag1*^*−/−*^ mice were added. (E) *CNS1*^*−/−*^ mice were fed with either LF or WF for 2 weeks and their colonic lamina propria Tregs were analyzed by flow cytometry. Results are representative of two independent experiments. Statistical significance was assessed using unpaired two-tailed *t* test (A and E) or one-way ANOVA followed by Sidak’s multiple comparisons test (B, C, and D). ***P < 0.001,****P < 0.0001.

The results outlined above led us to hypothesize that feeding WF-enriched diets to *Rag1*^*−/−*^ mice resulted in an environment that favored transplanted naïve T-cells becoming Tregs. In thinking about such an environment, we first hypothesized a role for CD206^+^ “M2-like” macrophages, which are known to promote Tregs and are increased by WF in WT mice.^8^ However, in contrast to WT mice, colonic levels of F4/80^+^CD206^+^ cells (i.e. M2-like macrophages) were not increased in response to WF in *Rag1*^*−/−*^ mice (Fig. 4A), potentially reflecting alterations in innate immunity and/or microbiota in *Rag1*^*−/−*^ mice but in any case, arguing against a role for M2-like macrophage cells in mediating the WF-induced increase in Tregs. Hence, we next considered the possibility that WF feeding generated colonic metabolites that acted on naïve CD4 T-cells to favor Treg development. We investigated this possibility via an in vitro assay wherein naïve CD4^+^ T cells were incubated with dendritic cells (DC), soluble anti-CD3, Treg-promoting cytokines (IL-2 and TGFβ), and fecal supernatants (FS) from mice fed GBC, LF or WF diets. Addition of WF FS resulted in higher levels of Tregs compared to GBC and LF FS, albeit less than that observed in response to TGFβ positive control (Fig. 4B). To discern the extent to which FS had acted upon the DC or T cells, DC were replaced with anti-CD28 and plate-bound anti-CD3. A similar pattern of results was observed, arguing that WF FS had resulted in greater Treg levels via its action on T cells (Fig. 4C). The in vitro differentiation assay we utilized is thought to yield cells reminiscent of peripheral Tregs, whose development is dependent upon conserved non-coding sequence 1 knock-out (*CNS1*).^16^ Accordingly, we predicted WF would not promote Treg development in *CNS1*^*−/−*^ mice. Consistent with our expectation, naïve CD4^+^ T-cells from *CNS1*^*−/−*^ mice failed to develop into Tregs in vitro, irrespective of WF FS (Fig. 4D). Furthermore, in contrast to the case for WT mice, LF- and WF-fed *CNS1*^*−/−*^ mice, which harbor only thymic Tregs, did not differ levels of colonic Tregs supporting our presumption that WF was promoting Treg development in the periphery (Fig. 4E).

Our study of WF’s impact on acute colitis revealed that catabolism of WF by *Bacteroides thetaiotaomicron* (*B. theta*), which is rich in carbohydrate-active enzymes, resulted in elevated fecal levels of a polyphenol, namely isofraxidin, that partially recapitulated the impact of WF to alter macrophage polarity and protect against DSS-induced colitis.^8^ This led us to hypothesize that isofraxidin might promote Tregs, but we found that it failed to do so, in vitro, over the wide range of concentration tested (Supplementary Fig. 4A). Hence, we probed the more general hypothesis that other products of WF catabolism might promote Tregs. In accord with this notion, fecal supernatants of ASF/*B. theta* colonized mice fed WF, but not LF, promoted Treg development in vitro. We leveraged our earlier observations that isofraxidin, identified via fecal metabolomics, was upregulated by WF when ASF mice were colonized with *Bacteroides thetaiotaomicron* (*B. theta*). The fecal supernatant of such mice induced higher Tregs frequencies at higher concentration (Supplementary Fig. 4B). Still, culture supernatant of *B. theta* grown in the presence of WF induced greater Treg differentiation (Supplementary Fig. 4C), suggesting WF/*B. theta-*derived metabolites may promote Treg differentiation. Yet, unlike our sister study, *B. theta* was not detected in the *Rag1*^*−/−*^ mice vivarium (Fig. 3C and Supplementary Fig. 1C); therefore we view *B. theta* as a model bacterium illustrating a potential mechanism.

**Fig. 5.**
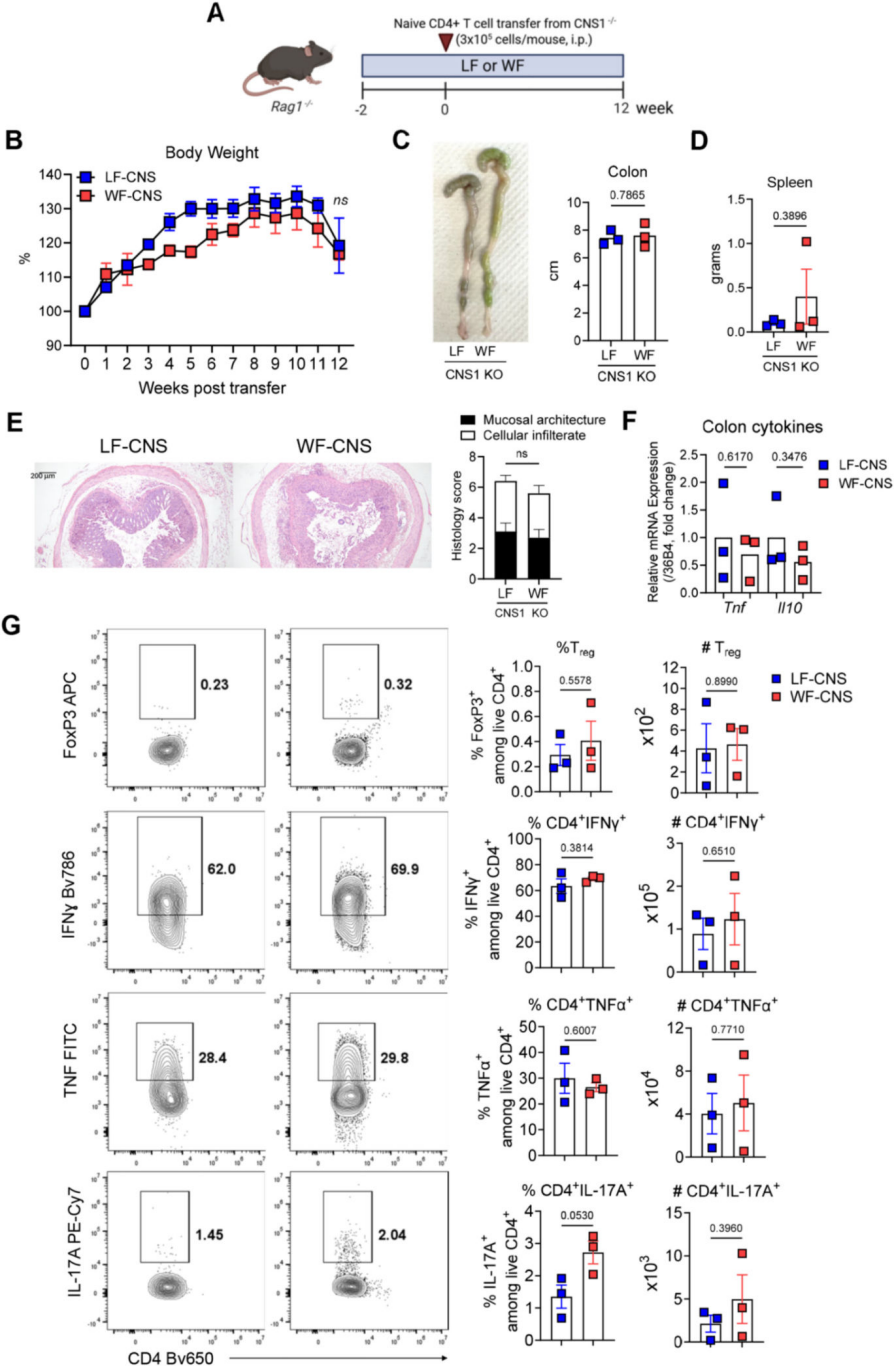
Wheat fiber did not protect against T cell colitis in the absence of peripheral regulatory T cells. *Rag1*^*−/−*^ mice (n = 3 per group) were fed either LF or WF for 2 weeks and received splenic 3×10^5^ naïve CD4^+^ T cells from *CNS1*^*−/−*^ mice. (A) Body weight. (B) Spleen weight. (D) Colon length. (E) Colon inflammatory cytokine levels measured by RT-qPCR. (F) Representative colon histology and scoring. (G) Colonic lamina propria CD4^+^ T cells analyzed by flow cytometry. Results are representative of two independent experiments. Statistical significance was assessed using Student’s *t* test or two-way ANOVA followed by Sidak’s multiple comparisons test.

Lastly, we probed the role of peripheral Treg development in mediating WF’s protection against T-cell-mediated colitis. CD4^+^CD25^−^CD45Rb^hi^ (i.e. naïve) T-cells, isolated from GBC-fed *CNS1*^*−/−*^ mice, were transplanted into *Rag1*^*−/−*^ mice consuming LF or WF diets (Fig. 5). Both groups of mice began to lose weight 10 weeks post-transfer and did so with similar kinetics, suggesting a lack of protection by WF. Post-mortem assessment of colitis, based on morphology, histopathology, and cytokine analysis also indicated that WF did not protect against colitis driven by *CNS1*^*−/−*^ T-cells. Furthermore, WF feeding did not result in increased levels of colonic lamina propria-derived FoxP3^+^ cells, nor did it decrease levels of IFNɣ^+^, TNFα^+^, or Il-17A^+^ CD4 T cells. Thus, the ability of WF feeding to promote Tregs, suppress colitogenic T-cells, and protect mice against T-cell mediated colitis required *CNS1*.

## Discussion

The post-mid-20th century increase in the prevalence of IBD may have been promoted by changes in diet that have impacted the host-microbiota relationship. One example of an observation that supports this overarching hypothesis is our recently reported finding that wheat fiber (WF), a traditional major component of wheat-based foods but largely absent in refined flour, helps mice maintain select microbial taxa that dampen pro-inflammatory signaling in intestinal macrophages, thereby ameliorating severity of acute, chemical-induced, colitis.^8^ The goal of this study was to define the extent to which WF impacted the severity of T-cell mediated colitis and investigate underlying mechanisms. We found that, indeed, WF protected mice in a well-characterized model of T-cell transfer colitis. Such protection was mediated by pTregs, which were induced in a microbiota-dependent manner by WF. These results suggest that the reduction in WF consumption that resulted from industrialization is one factor that may have contributed to the increases in chronic T-cell mediated diseases, including IBD, and that, regardless, consumption of WF may reduce risk for developing such diseases.

The notion that WF’s protection against T cell mediated colitis was mediated by pTregs arose from our observation that these cells were induced by WF and was confirmed by the failure of WF to protect recipients of CNS1-null T cells from colitis. These results comport with earlier findings that pTreg defects, resulting from Treg-specific deletion of RORγt, exacerbate the severity of T-cell-mediated colitis^14,17,18^ and the general notion that dietary fiber promotes their differentiation. While our study focused on deciphering how WF drove Tregs, we were surprised to observe that naïve T cells from CNS1-mull mice appeared to drive colitis more slowly than cognate cells from WT mice. Future studies will be needed to explain this counter-intuitive result. The best-characterized means by which fiber is reported to promote Tregs is via its fermentation product, butyrate. Indeed, administering mice arabinoxylan, a soluble component of some WF, enhances butyrate production and Treg differentiation, thereby conferring protection in this adoptive transfer model of colitis.^19^ However, the food-grade WF used in this study is largely insoluble, contains minimal arabinoxylan and, accordingly, did not increase butyrate levels,^8^ arguing against a role for short-chain fatty acids. A diverse array of dietary fibers, including inulin and psyllium, increase serum bile acids (BA),^4,7^ which can promote Tregs,^20^ suggesting another means by which dietary fiber increases these cells. However, that BA promote Tregs in vitro in a DC-dependent manner^20^ while WF resulted in higher Tregs in vitro irrespective of DC argued against a role for BA. Furthermore, we observed that levels of total serum BA were similar in LF- and WF-fed mice arguing against this possibility. Thus, the mechanism by which WF-feeding resulted in increased Tregs is not yet clear. Our results from the in vitro Treg assay suggested that differences in the intestinal metabolome between LF- and WF-fed mice were responsible for the differences in Tregs observed in vitro but whether this reflected the WF FS actually contained molecules that acted on T-cells to promote Treg development or simply contained less of a factor that inhibited Treg development could not be discerned in our experiments.

WF failed to induce Tregs in ASF mice, which harbor a minimal microbiota. WF did not alter microbiota density, suggesting a potential contribution from WF-induced changes in microbiota composition in driving Tregs. WF induced changes increases in several species, including *A. muciniphilia*, which expresses epitopes capable of inducing Tregs.^21^ Furthermore, abundance of this bacteria is low in IBD patients^22^ and it has been correlated with protective roles in IBD.^23^ WF also increased *Clostridium ruminantium* which is typically found in ruminants^24^ and has not yet been reported in the murine gut. The abundance of *Clostridium celatum* was lowered by wheat fiber, and a similar trend was observed by a plant-derived anti-inflammatory flavonoid.^25,26^ Also, this bacteria was increased in the groups with diarrhea in immunocompromised mice^27^ and celiac disease patients.^28^ Future studies to investigate the role of these taxonomic changes and, more generally, better understand how WF feeding promotes Treg development are warranted.

Our recent observation that WF ameliorates DSS colitis led us to suggest that its consumption by humans might reduce the severity of disease flares of IBD patients.^8^ Our results herein suggest that consumption of whole wheat foods, especially as one’s immune system develops in childhood, when naïve T cells are exposed to different stimuli, might reduce future risk of developing an array of immune-mediated chronic inflammatory diseases including IBD. In contrast, feeding mice a WF-enriched, rather than the LF diet, as symptoms and inflammatory indices of T-cell colitis began manifesting, did not alter the course of disease development. This may reflect that the pro-inflammatory environment impeded WF-induced promotion of Tregs and/or their inability to constrain activated T-cells that predominated at this stage of the disease model. Alternatively, it may be that the intervention was simply begun to late and thus may have potential to slow colitis if initiated earlier in disease development. In any case, our results accord with observations in humans that dietary modifications are unlikely to lastingly cure IBD but may serve to prevent and/or delay its onset.

## Methods

### Mice and diets

C57BL/6 wild-type (WT) and *Rag1*^*−/−*^ were purchased from Jackson Laboratories (Bar Harbor, ME) and Conserved Non-coding Sequence 1 knockout (*CNS1*^*−/−*^) mice were provided by Alexander Rudensky (Sloan Kettering Institute). Gnotobiotic mice with ASF were generated from C57BL/6 germ-free mice purchased from Taconic Biosciences Inc. (Rensselaer, NY). All mice were bred/housed at Georgia State University under Institutional Animal Care and Use Committee #A20043 and #A24001. Initial experiments included both male and female mice, but no sex-specific differences were observed. Consequently, female mice were used for all subsequent experiments. All mice were maintained under normal grain-based chow (GBC) until the indicated experimental diets: a low-fiber diet (LF), containing only 50 g of cellulose as dietary fiber, or LF enriched with 150 g of purified wheat fiber. Detailed diet compositions are described in Table 1.

### Adoptive T cell transfer model of colitis

Nine-week-old *Rag1*^*−/−*^ mice were administered FACS-sorted CD4^+^CD25^-^CD45Rb^hi^ T cells (2–3×10^5^ cells/mouse) via intraperitoneal injection.^29^ Such naïve T cells were isolated from spleens of either C57BL/6 wt mice or *CNS1*^*−/−*^ mice as indicated. After the transfer, the recipient mice were monitored weekly for weight loss and diarrhea and euthanized when mice lost > 20% of body weight on the day of transfer. Disease activity index was measured on the day of sacrifice according to the criteria as previously described.^8^ .

### Isolation of lymphocytes from colonic lamina propria

Dissected colons were cut into small pieces and washed with calcium- and magnesium-free Hanks’ balanced salt solution (CMF/HBSS). The epithelium was removed by two consecutive incubation in a shaker (200 rpm, 20 min, 37 ^◦^C) with 2 mmol/L ethylenediaminetetraacetic acid (EDTA) in CMF/HBSS containing 5% fetal bovine serum. Intestinal tissue pieces were digested with 1 mg/ml of collagenase type IV (Sigma C5138) and 40 μg/ml of DNase I (Roche, Germany) in CMF/HBSS or RPMI containing 5% fetal bovine serum at 37 ^◦^C, 150 rpm, for 10–12 min. Cells were then filtered (40 μm), centrifuged, and supernatants were discarded and resuspended in cold CMF/HBSS. Lamina propria mononuclear cells were further purified by Percoll (GE Health Care, Uppsala, Sweden) density gradient centrifugation.^8^ .

### In vitro differentiation of regulatory T cells

Naïve CD4^+^T cells and CD11c^+^dendritic cells were isolated from the spleens of wild-type (WT) mice using the MACS purification kit (Miltenyi Biotec, #130–104–453 or #130–125–835) following the manufacturer’s instructions. Purified naïve CD4^+^ T cells (5 × 10^4^ cells/well) were then cultured, with or without dendritic cells (10^4^ cells/well) for three days in T cell medium (RPMI, 10% fetal bovine serum, 1% HEPES, 1% nonessential amino acids, 1 mM sodium pyruvate, 55 μM 2-mercaptoethanol, 100 U/ml penicillin, 100 μg/ml streptomycin). To induce FoxP3^+^ regulatory T cells, anti-CD3 antibodies (2.5 μg/mL), recombinant human IL-2 (15 U/mL), and TGF-β (1 ng/mL) were added.^20^ For the dendritic cells-free assay, a 96-well cell culture plate was coated with anti-CD3 antibodies (2.5 μg/mL), and anti-CD28 (1 μg/mL) antibodies were added instead of dendritic cells. Fecal supernatants (100 mg/ml in PBS) were added (1:50 dilution or indicated) on the same day when naïve T cells were seeded as indicated in the corresponding figures.

### Flow cytometry

Cells were first blocked with 1 μg per million cells of 2.4G2 (Bio-XCell) to prevent Fc receptor-mediated binding. Live cells were identified by excluding dead cells using the Fixable Aqua Dead Cell Staining Kit (Invitrogen). After washing with PBS, cells were stained for surface markers (i.e., CD4) using conjugated monoclonal antibodies (mAbs) in FACS buffer. For intracellular staining, cells were re-stimulated with 1X Cell Activation Cocktail with Brefeldin A (BioLegend) for 3 h, followed by fixation and permeabilization using the Fixation/Permeabilization (Fix/Perm) Buffer Set (eBioscience). Subsequently, cells were stained for inflammatory cytokines and FoxP3 in the FoxP3 Staining Buffer (eBioscience).^8^ Fluorochrome-conjugated mAbs were used as indicated in the figures. Multi-parameter flow cytometry was performed on a CytoFlex (Beckman Coulter) and analyzed using FlowJo software (Tree Star). Cell sorting was conducted on a SONY SH800Z cell sorter (Sony).

### RNA extraction and qRT-PCR

Total RNA was extracted from colons using Trizol (Invitrogen, Carlsbad, CA) according to the manufacturer’s protocol. Quantitative RT-PCR was performed using iTaq™ One-Step RT-PCR Kit with SYBR Green (Bio-Rad, Hercules, CA) in a CFX 96 apparatus (Bio-Rad, Hercules, CA). using the following primers (F/R): *36b4*: 5′-TCCAGGCTTTGGGCATCA-3′, 5′-CTTTATTCAGCTGCACATCACTCAGA-3′; *Tnf*: 5′-CGAGTGACAAGCCTGTAGCC-3′, 5′-CATGCCGTTGGCCAGGA-3′; *Il10*: 5′-CAGTACAGCCGGGAAGACAA-3′, 5′-GGCTTGGCAACCCAAGTAA-3′. Differences in transcript levels were normalized to the housekeeping gene *36b4.*

### Histopathologic analysis

Fresh colons were fixed in 10% PBS-buffered formalin. Paraffin embedding, sectioning, and hematoxylin and eosin (H&E) staining were performed at HistoWiz, Inc. The stained slides were blindly scored by two pathologists, Shawn Winer. and Daniel A. Winer as previously described.^4^ .

### Bacteria quantification in feces by qPCR

To measure the total fecal bacterial load, total DNA was isolated from weighted feces using QIAamp DNA Stool MiniKit (Qiagen, Hilden, Germany). DNA was then subjected to qPCR using QuantiFast SYBR Green PCR kit (Bio-Rad, Hercules, CA) with universal 16S rRNA primers 8F: 5′-AGAGTTTGATCCTGGCTCAG-3′ and 338R: 5′-CTGCTGCCTCCCGTAGGAGT-3′ to measure total bacteria number. Results are expressed as bacteria number per mg of stool using a standard curve.^4^ .

### Lipocalin-2 ELISA

Fecal lipocalin-2 levels were measured using DuoSet mouse lipocalin-2 ELISA kit (R&D Systems, #DY1857) according to the protocol.^9^ Fecal supernatants were collected after centrifuging (12,000 rpm, 10 min, 4 ^◦^C) the homogenized feces (100 mg of feces/ml of PBS).

### Gut microbiota analysis by 16S rRNA gene sequencing

Fecal DNA was extracted using DNeasy 96 PowerSoil Pro QIAcube HT Kit (Qiagen), and the region V3-V4 of 16S rRNA genes were amplified using the following primers: 341F 5′-TCGTCGGCAGCGTCAGATGTGTATAAGAGACAGCCTACGGGNGGCWGCAG-3′; 805R 5′-GTCTCGTGGGCTCGGAGATGTGTATAAGAGA-CAGGACTACHVGGGTATCTAATCC-3′. PCR products of each sample were purified using Ampure XP magnetic purification beads. An index PCR was performed to attach dual barcodes and Illumina sequencing adapters using Nextera XT Index kit (Illumina). Final PCR products were verified on 1.5% DNA agarose gel and quantified using Pico dsDNA assay (Invitrogen). An equal molar of each sample was combined and purified again using Ampure XP beads as the library. The library was diluted and spiked with 5% PhiX control (Illumina) and sequenced by Illumina MiSeq sequencing system (2 × 250 bp). Demultiplexed fastq files were generated on instrument. Sequence reads were quality-filtered by DADA2 plugin in Qiime2.^30^ Taxonomy was assigned based on the Greengenes database. Raw sequencing data have been deposited at NBCI Sequence Read Archive (https://www.ncbi.nlm.nih.gov/sra): PRJNA1250494.

### Statistical analysis

GraphPad Prism 10.0 was used for all statistical testing. All data are given as mean ± SEM, and were compared via unpaired two-tailed *t* test or one-way or two-way ANOVA followed by Tukey’s multiple comparison test or Sidak’s multiple comparison test. Significance is expressed as *p < 0.05, **p < 0.01, ***p < 0.001, ****p < 0.0001.

## Supplementary Material

1

## Figures and Tables

**Table 1:** Diet compositions.

	LF		WF		Cellulose	
Research Diets Cat#	D12450J		D20030503		D13081109	
	50 g Cellulose Only		50 g Cellulose. 150 g Wheat Fiber		200 g Cellulose	
	gm%	kcal%	gm%	kcal%	gm%	kcal%
Protein	19	20	17	20	17	20
Carbohydrate	67	70	59	70	59	70
Corn Starch	48	50	42	50	42	50
Fat	4	10	4	10	4	10
**Total**		100		100		100
**Ingredient**	**gm**	**kcal**	**gm**	**kcal**	**gm**	**kcal**
Casein	200	800	200	800	200	800
L-Cystine	3	12	3	12	3	12
Corn Starch	506.2	2025	506.2	2025	506.2	2025
Maltodextrin 10	125	500	125	500	125	500
Sucrose	68.8	275	68.8	275	68.8	275
Cellulose, BW200	50	0	50	0	200	0
Wheat Fiber 600, Vitacel	0	0	150	0	0	0
Lard	20	180	20	180	20	180
Soybean Oil	25	225	25	225	25	225
Mineral Mix S10026	10	0	10	0	10	0
						
Dicalcium Phosphate	13	0	13	0	13	0
Calcium Carbonate	5.5	0	5.5	0	5.5	0
Potassium Citrate, 1 H2O	16.5	0	16.5	0	16.5	0
Vitamin Mix V10001	10	40	10	40	10	40
Choline Bitartrate	2	0	2	0	2	0
Yellow Dye #5, FD&C	0.04	0	0.01	0	0	0
Blue Dye #1, FD&C	0.01	0	0.04	0	0.05	0
**Total**	1055.05	4057	1205.05	4057	1205.05	4057
**kcal/gm**	3.85			3.37		
	**gm**	**gm%**	**gm**	**gm%**	**gm**	**gm%**
**Total Fiber**	50	4.7	189.1	15.7	200	16.6
**Insoluble Fiber**	50	4.7	185.2	15.4	200	16.6
**Soluble Fiber**	0	0	3.9	0.3	0	0

## Appendix A. Supplementary material

Supplementary data to this article can be found online at https://doi.org/10.1016/j.mucimm.2025.12.003.
