# Supplementary material for: Wheat fiber-induced peripheral regulatory T-cells suppress development of colitis
Source: Mucosal Immunol. Author manuscript; Available in PMC 2026 Jun 17. (PMC13274533; doi:10.1016/j.mucimm.2025.12.003)
Supplement: 1 [file NIHMS2179686-supplement-1.pdf]

Supplementary Figures

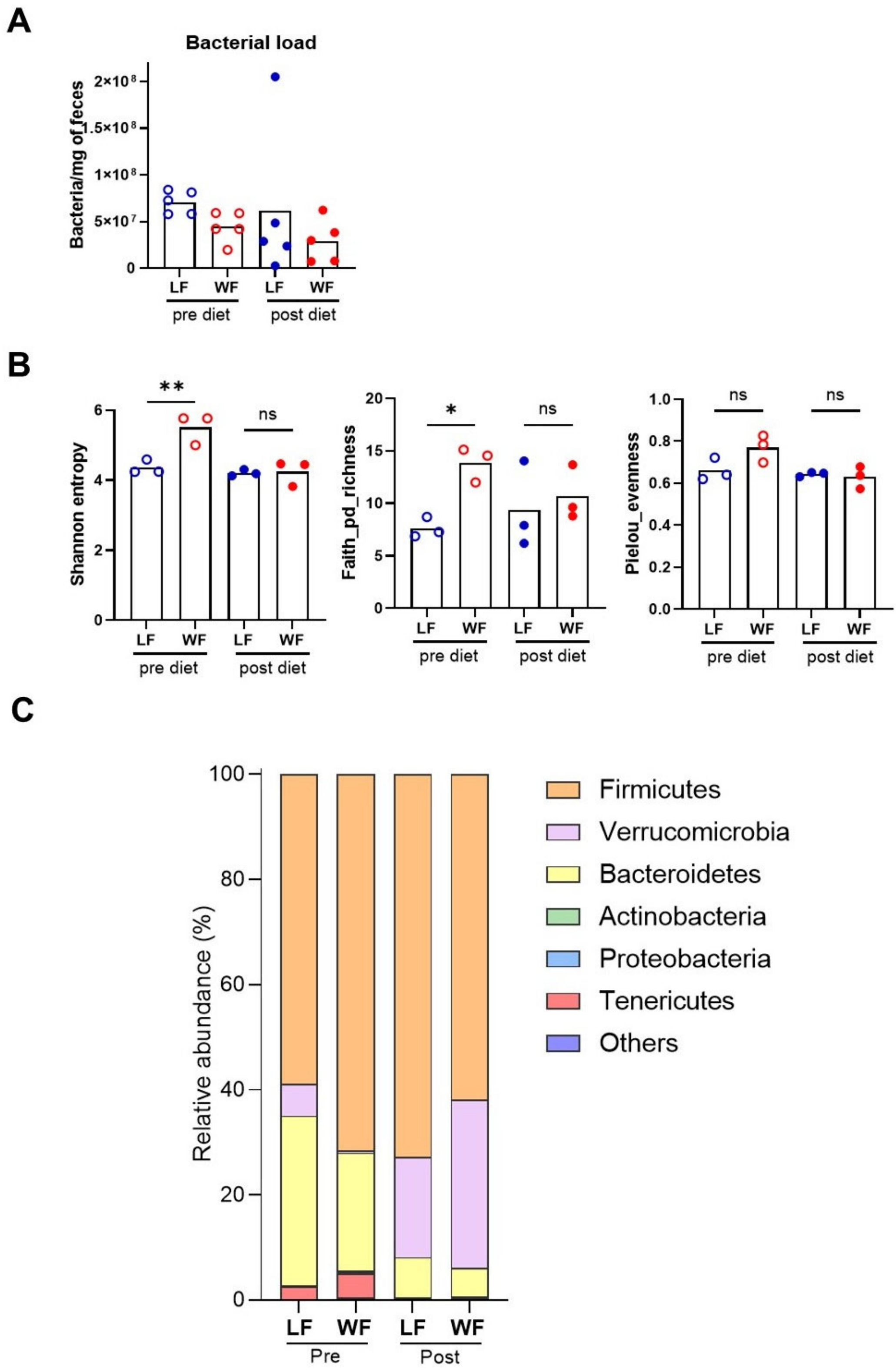

Supplementary Figure 1. Fecal bacterial analysis of *Rag1$^{-/-}$* mice on low-fiber or wheat fiber diets.

Rag1$^{-/-}$ mice were fed either LF or WF for 2 weeks and their feces were analyzed.

(A) Total bacterial load measured by qPCR with 16S rRNA primers. (B) Alpha diversity index analyzed by MiSeq 16S sequencing. (C) Taxonomic composition at the phylum level. Statistical significance was assessed using one-way ANOVA followed by Sidak's multiple comparisons test. *$P < 0.05$, **$P < 0.01$.

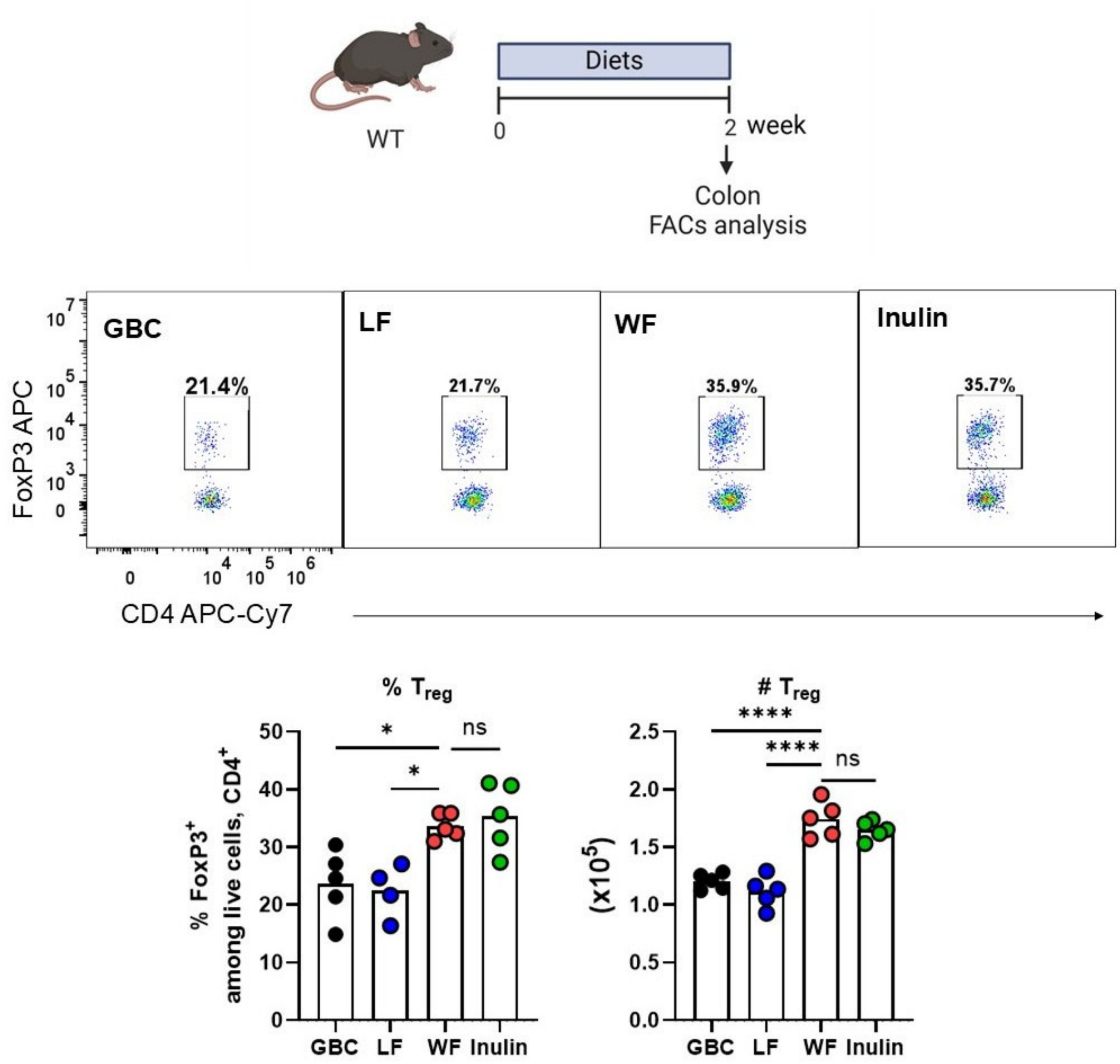

Supplementary Figure 2. Wheat fiber's induction of Tregs is comparable to that of fermentable fiber, inulin.

WT conventional mice (n=5/group) were fed with GBC, LF, WF, or Inulin for 2 weeks and their colonic lamina propria Tregs were analyzed by flow cytometry. Results are representative of two independent experiments. Statistical significance was assessed using one-way ANOVA followed by Tukey's multiple comparisons test. *$P < 0.05$,****$P < 0.0001$.

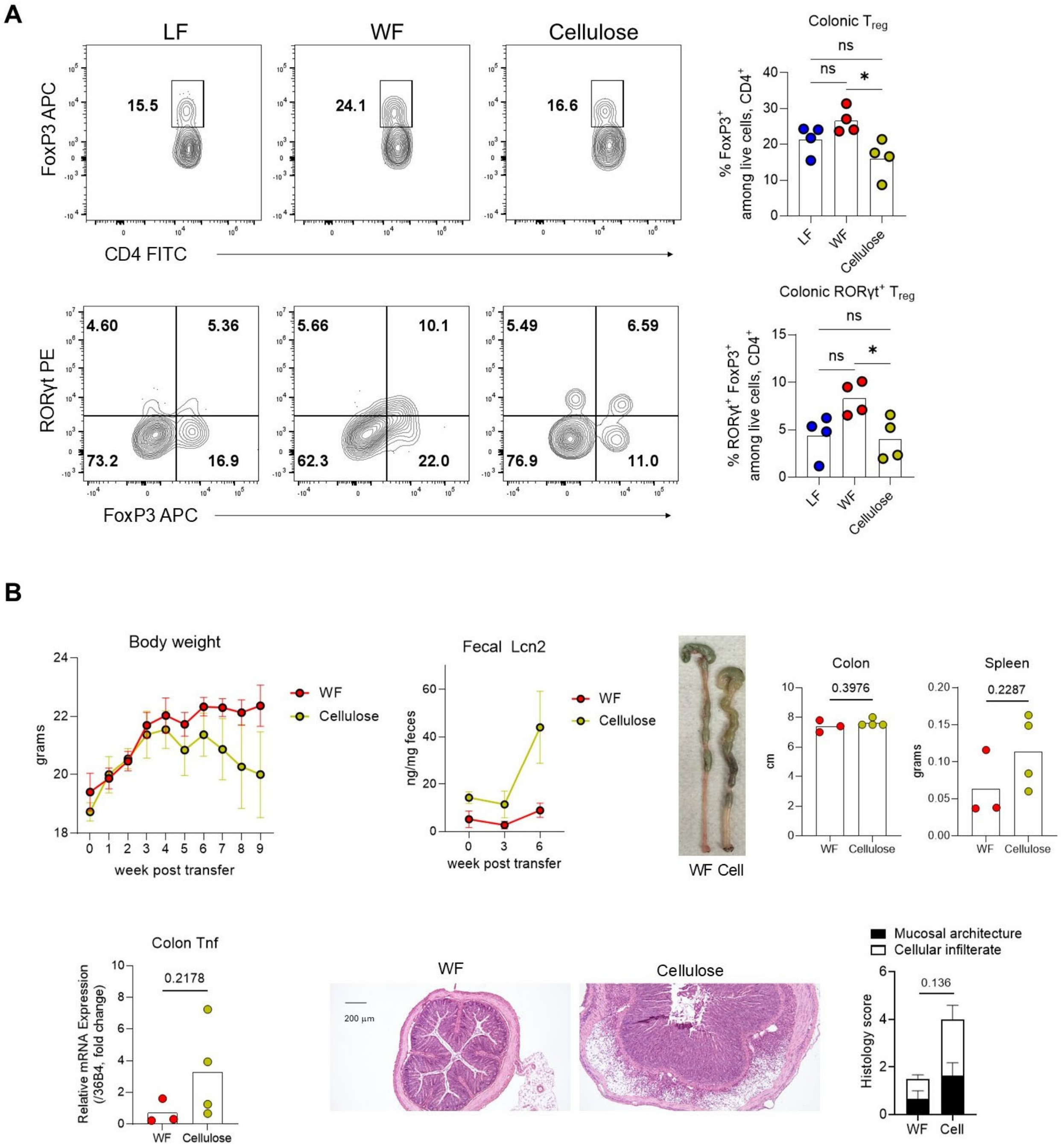

Supplementary Figure 3. Cellulose failed to induce colonic Tregs nor protect mice against T-cell-mediated colitis.

(A) WT conventional mice (n=4/group) were fed with LF, WF, or Cellulose for 2 weeks and their colonic lamina propria Tregs were analyzed by flow cytometry.

(B) *Rag1*$^{-/-}$ mice (n=3-4/group) were fed with LF, WF, or Cellulose for 2 weeks and naïve T cells from WT mice were transferred to induce T-cell-mediated colitis. Weight loss and fecal Lcn2, colon length, spleen weight, colonic Tnf transcript, and histology were measured.

Statistical significance was assessed using one-way ANOVA followed by Tukey's multiple comparisons test or unpaired two-tailed t test. ns $P > 0.05$, $^{*}P < 0.05$.

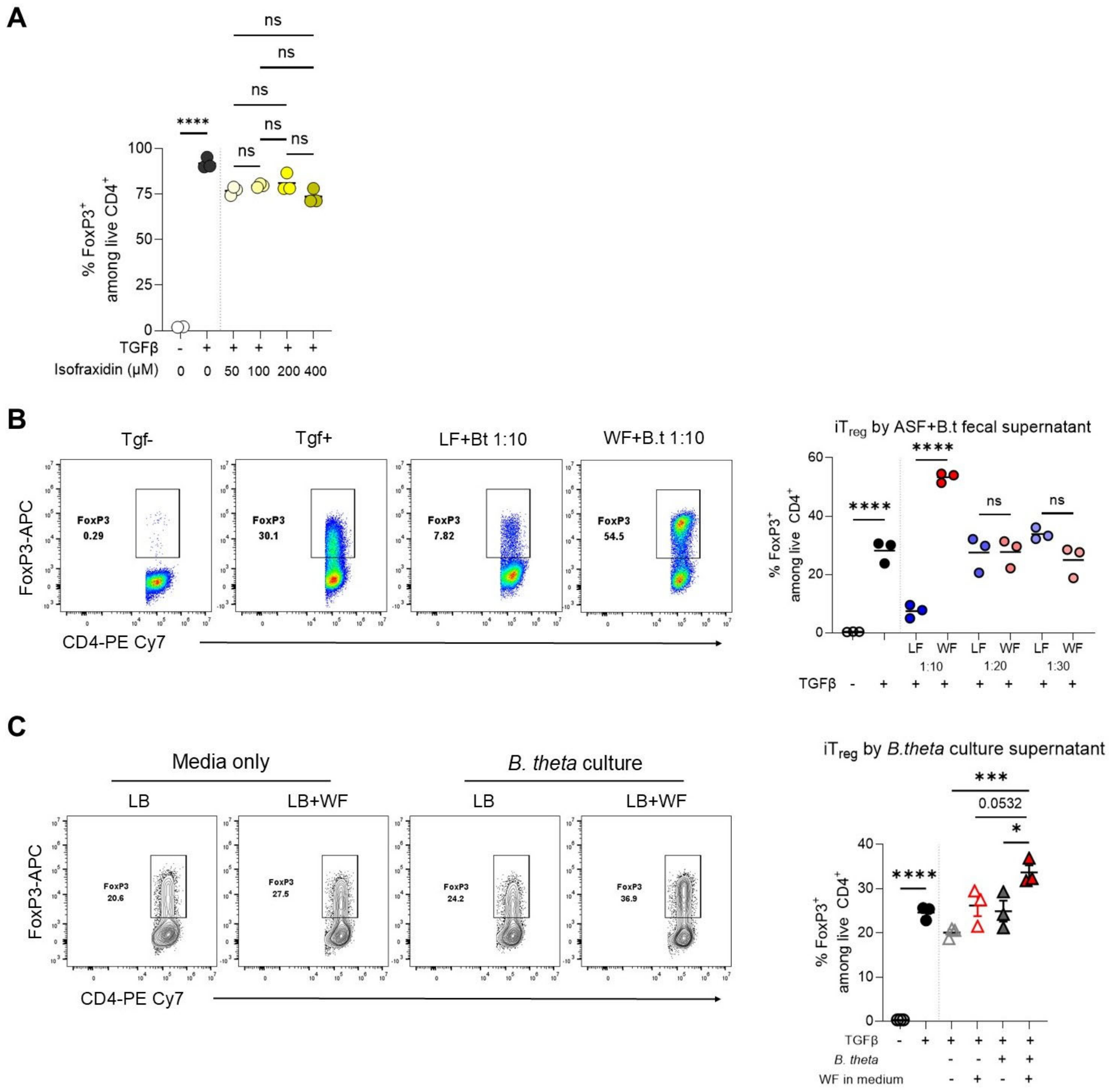

Supplementary Figure 4. *Bacteroides thetaiotaomicron*-related metabolites tested on Treg induction.

Naïve CD4+ T cells (0.5~1x10^5 cells/well) from WT mice were cultured on aCD3ε-coated (2 μg/ml) plates with αCD28 (2.5 μg/ml), IL-2 (15 U/ml), and TGF-β (1 ng/ml). Isofraxidin (A), fecal supernatants from LF or WF-fed ASF mice colonized with *Bacteroides thetaiotaomicron* (*B. theta*) (B), or *B. theta* culture grown in half LB media with or without wheat fiber (1:25

dilution) (C) was added on day 0. On day 3, induced $FoxP3^+$ Tregs were analyzed by flow cytometry.

Statistical significance was assessed using one-way ANOVA followed by Tukey's multiple comparisons test. ns $P > 0.05$, ***$P < 0.001$, ****$P<0.0001$
